# Posterior urethroplasty for pelvic fracture urethral injuries: risk factors for recurrence and complications

**DOI:** 10.1007/s00345-025-05839-3

**Published:** 2025-08-01

**Authors:** Natalia Plamadeala, Marjan Waterloos, Mieke Waterschoot, Nicolaas Lumen

**Affiliations:** 1 Department of Urology, Ghent University Hospital, Ghent, Belgium; 2 Department of Urology, Department of Surgical Sciences, A.O.U. Città della Salute e della Scienza, University of Turin, Turin, Italy; 3 Department of Urology, AZ Maria Middelares, Ghent, Belgium

**Keywords:** Posterior urethroplasty, Pelvic fracture urethral injury, Urethral reconstruction, Stenosis recurrence, Urethroplasty complications

## Abstract

**Purpose:**

Surgical reconstruction for posterior urethral injuries associated with pelvic fractures (PFUI) is complex. Perineal posterior urethroplasty via excision and primary anastomosis (EPA) is the standard treatment. This study evaluates risk factors for stenosis recurrence after primary EPA and analyzes predictors of postoperative complications.

**Methods:**

A retrospective analysis was conducted on male patients who underwent primary posterior EPA for PFUI at Ghent University Hospital (2001–2024). The primary outcome was stenosis recurrence at 1-, 2-, and 10-year follow-up, defined as urethral narrowing causing obstructive symptoms and requiring intervention. Demographic data, prior urethral manipulation, surgical techniques, 90-day complications (Clavien-Dindo), and functional outcomes were analyzed. Univariate and multivariate coxregression analyses were performed using IBM SPSS Statistics v. 29.0.2.0.

**Results:**

Among 70 patients, 75.5% underwent transecting EPA. After a median follow-up of 130 months (IQR 78.5-178.5), stenosis recurrence occurred in 15.8% at a median of 3 months (IQR 2.0–5.0). The 1-, 2-, and 10-year recurrence-free survival rates were 85.4%, 83.8%, and 83.8%, respectively. In multivariate analysis, postoperative complications (HR = 4.85, *p* = 0.007) and persistent extravasation (HR = 6.36, *p* = 0.006) significantly increased recurrence risk. Postoperative complications (21.4%) were all low-grade and managed conservatively. Erectile function was impaired in 97.9% due to trauma, with 12.2% improving postoperatively. De novo urinary incontinence occurred in 6.6%.

**Conclusions:**

Posterior urethroplasty demonstrates a high long-term success rate in patients with PFUI. Postoperative complications and persistent urinary leakage significantly increase the risk of surgical failure, highlighting the need for rigorous follow-up. The non-transecting technique, when possible, does not negatively impact outcomes.

**Supplementary Information:**

The online version contains supplementary material available at 10.1007/s00345-025-05839-3.

## Introduction

A pelvic fracture can be associated with urethral injury in 1.6–25% of cases, with urethral rupture being more common in men than in women [1]. Such injuries can lead to long-term sequelae, including urethral stenosis, erectile dysfunction (ED), and urinary incontinence (UI), significantly impacting quality of life [2–5].

Urethroplasty by excision and primary anastomosis (EPA) is the standard surgical approach for obliterative stenosis of the posterior urethra following male pelvic fracture urethral injury (PFUI), also used in non-obliterative stenosis as a first-line treatment or after failed primary endoluminal interventions [6–8].

The acute management of PFUI remains controversial. In the acute setting, initial management focuses on hemodynamic stabilization and urinary diversion to prevent extravasation and infection in cases of suspected urethral rupture. Some studies suggest that primary realignment may reduce stenosis formation compared to suprapubic tube (SPT) placement with delayed EPA, while others highlight potential drawbacks of early endoscopic realignment [9–12].

Delayed posterior urethroplasty remains the gold standard for managing PFUI, allowing hematoma resolution, prostate descent, scar tissue stabilization, and optimal patient positioning [13].

In EPA, scar tissue between the distracted urethral ends is excised, and a tension-free anastomosis is performed. If the bulbar urethra cannot be mobilized sufficiently to bridge the gap, additional manoeuvres such as corporeal separation, inferior pubectomy, and supracrural rerouting are used in sequence [14–17].

Posterior urethroplasty has shown high success rate in managing PFUI-related stenosis, with reported success rates of up to 96% [3, 18, 19]. However, the risk of postoperative complications and recurrent stenosis remains a concern [19–21]. The definition of urethral stenosis recurrence varies across the literature, but it is most commonly considered as the need for reintervention. In our study, recurrence was defined as a re-narrowing at the urethroplasty site causing obstructive symptoms, confirmed by retrograde urethrography (RUG) or cystoscopy, and requiring re-intervention. Identifying factors associated with increased complication rates and EPA failure is crucial to optimize surgical outcomes and patient management.

This study aims to evaluate risk factors for stenosis recurrence following primary EPA urethroplasty and assess postoperative complication predictors.

## Materials & methods

### Study design, patients’ characteristics, and data collection

Following approval from the Institutional Review Board of UZ Ghent, Ghent University Hospital, (Protocol Number EC/UZG/2008/234), all males who underwent treatments for urethral stricture/stenosis at Ghent University Hospital, a tertiary referral center for urogenital trauma and reconstruction in Belgium, were retrospectively included in a database starting from June 2001, with prospective maintenance from 2008 onward. A total of 1,689 male patients who underwent any form of treatment for urethral stricture/stenosis at our center were recorded in the database between June 2001 and September 2024. This study was conducted following the ethical standards of the institutional and national research committees and the 1964 Helsinki Declaration and its later amendments, and all subjects provided written informed consent.

From this database, we identified 87 adult patients (aged ≥ 18 years) who underwent posterior urethroplasty for PFUI. Exclusion criteria included redo posterior urethroplasty, concomitant bladder neck stenosis requiring surgical intervention, incomplete clinical records, and non-traumatic causes of posterior urethral obstruction. As a result, 17 patients were excluded due to undergoing redo urethroplasty—3 of whom required it following recurrence after a primary procedure performed at our center, while the remaining patients were directly referred to us for redo urethroplasty after having had their primary surgery elsewhere. Consequently, this retrospective observational study includes 70 patients who underwent primary posterior urethroplasty for PFUI at our center.

The primary outcomes were the recurrent stenosis-free survival (RFS) rates and the reintervention rates for recurrent stenosis at 1-, 2-, and 10-year follow-up. Stenosis recurrence was defined as a re-narrowing at the urethroplasty site, causing obstructive urinary symptoms, confirmed by imaging (RUG) or urethroscopy (inability to pass a 16-Fr flexible cystoscope), thereby requiring further intervention such as dilation, urethrotomy, or re-do urethroplasty. The urethroscopy was reserved for cases in which RUG findings were inconclusive and patients presented with urinary complaints or a decrease in Qmax. The secondary outcomes aimed to describe and analyze patient demographics, surgical procedures, 90-day postoperative complications (using the Clavien–Dindo classification [22]), and functional outcomes related to erectile and urinary function following urethral reconstruction.

### Perioperative management

**Fig. 1:**
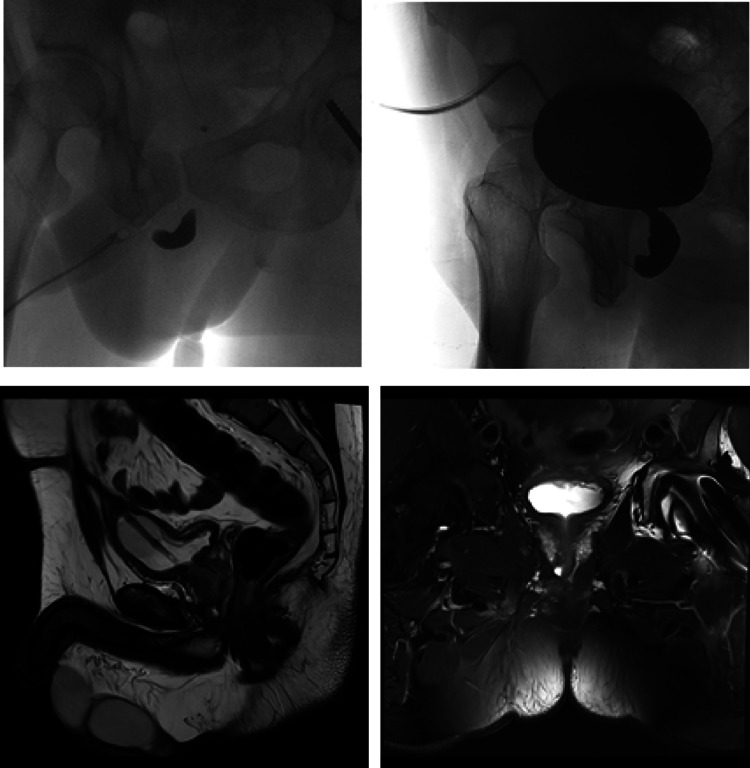
Preoperative retrograde urethrography, voiding cystourethrography, and MRI

All patients underwent preoperative RUG and voiding cystourethrography (VCUG) to assess the integrity of the anterior urethra, the length of the urethral defect, and the bladder neck preoperatively. In some cases, if the bladder neck did not open, the prostatic urethra could not be visualized, creating the impression of a much longer defect. In such situations, and when a double block was suspected, a pelvic MRI was performed to better evaluate the length of the distraction defect measuring the gap between the prostatic apex and the distal end of the urethral bulb, and to identify any periurethral abnormalities such as false passages, diverticula, or bone fragments (Fig. 1). Preoperative urinalysis was performed, and for patients with a positive urine culture (≥ 10³ CFU/mL), targeted antibiotic therapy was initiated 24 h before surgery based on antibiogram results and continued for at least five days postoperatively. However, in cases where multidrug-resistant organisms were identified, particularly *Pseudomonas aeruginosa* or ESBL producers, targeted antibiotic therapy was initiated at least five days prior to surgery, or according to the specific recommendations of an infectious disease specialist. In contrast, patients with a negative preoperative urinalysis received perioperative antibiotic prophylaxis with either 2 g of cephalosporin or 2.2 g of amoxicillin-clavulanic acid, administered intravenously.

**Fig. 2:**
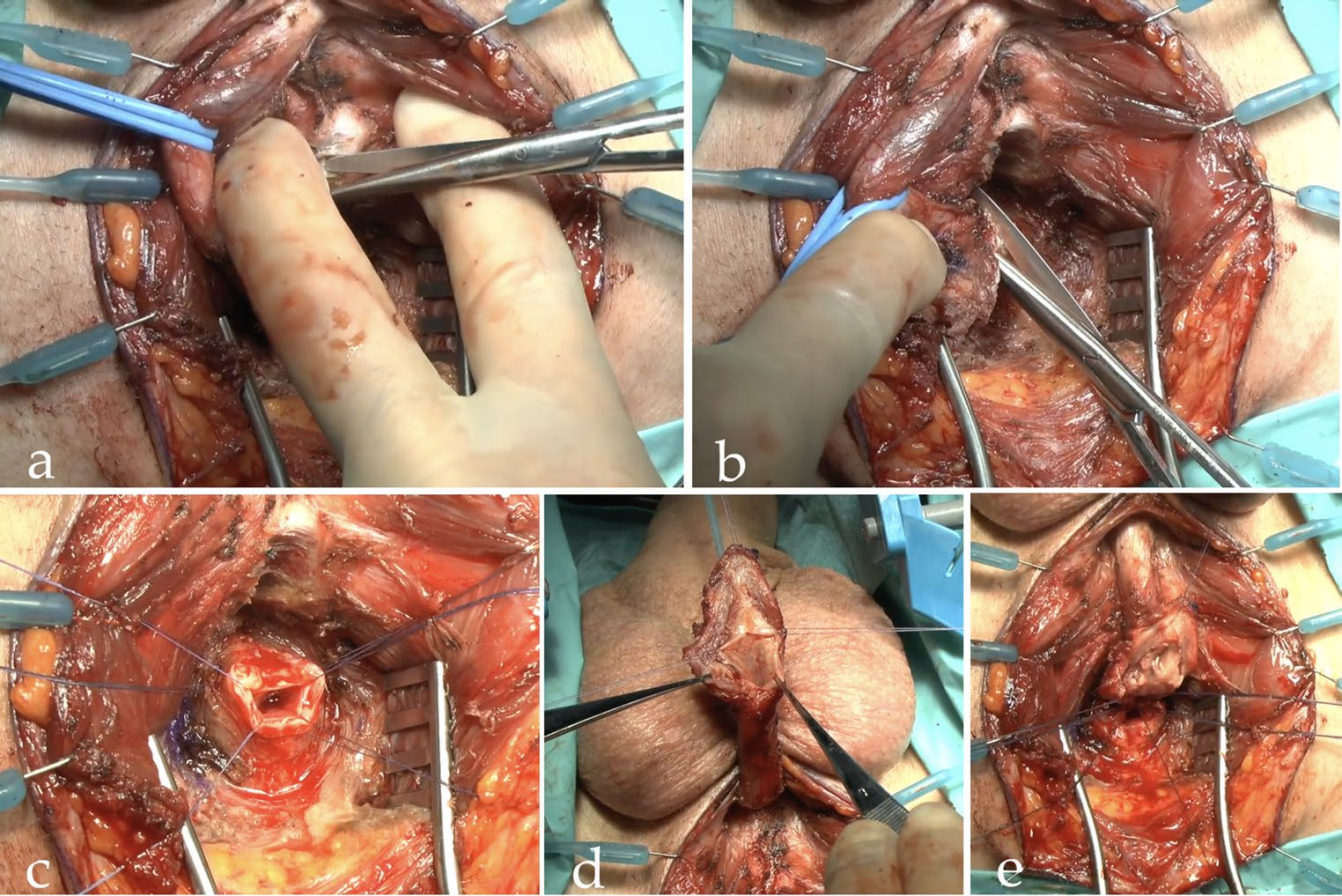
Transecting excision and primary anastomosis technique: **a** Splitting of the corpora cavernosa; **b** urethral transection; **c** spatulation of the proximal urethral end; **d** spatulation of the distal urethral end; **e** dorsal urethral plate anastomosis

**Fig. 3:**
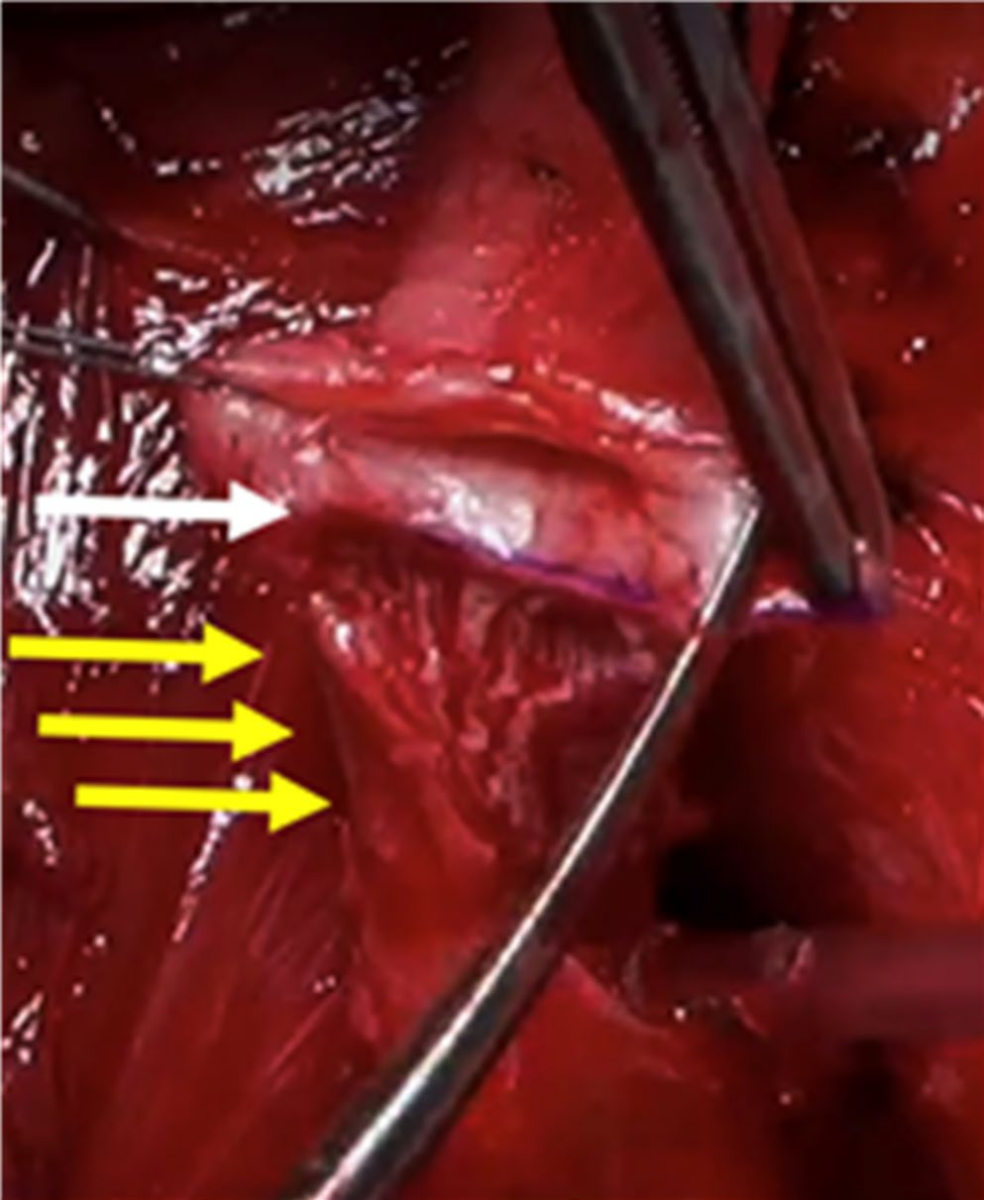
Vessel sparing (yellow arrow) in the non-transecting excision and primary anastomosis technique

**Fig. 4:**
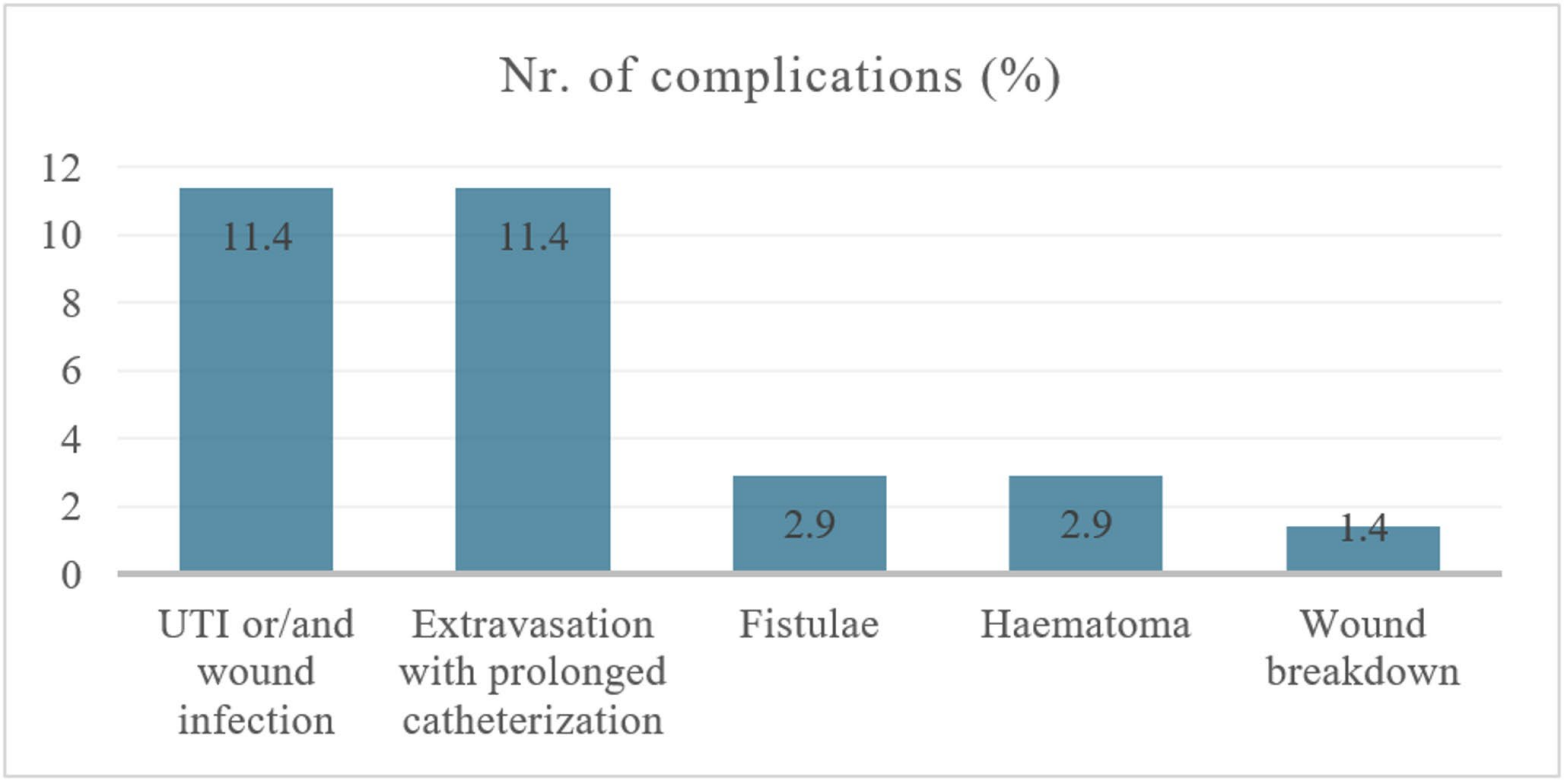
The overall number of complications in the study cohort (total of 18 complications, 25.7%)

ED and UI of any grade were assessed using a dichotomous (yes/no) patient-reported questionnaire before and after surgery. Assessing functional outcomes preoperatively in this population is inherently challenging due to factors such as the impact of pelvic trauma, suprapubic diversion, and reduced sexual activity. Patients were asked whether they experienced ED or UI following the trauma and prior to urethral surgery, as well as any changes at the 6-month follow-up after urethral reconstruction. This time point was chosen based on existing literature indicating that urethroplasty, particularly EPA for non-traumatic strictures, may cause transient erectile dysfunction, which typically resolves within 6 months. Beyond this period, recovery is unlikely, and outcomes are considered stable [23]. 

Following surgery, patients were seen for follow-up in an outpatient clinic, where they first underwent pericatheter retrograde urethrography (pRUG) approximately 14 days postoperatively. In cases of extravasation, pRUG was repeated weekly until resolution. Subsequent follow-ups were scheduled at 3, 9, 12, and 24 months, including uroflowmetry and ultrasound to assess post-void residual volume, with additional visits as needed. If patients reported urinary symptoms or showed a decrease in maximum flow rate (Qmax), further imaging, such as RUG, was performed at the physician’s discretion. Urethroscopy was not routinely performed during follow-up but reserved for cases where RUG results were inconclusive.

### Surgical procedure

We followed the standard approach, performing urethroplasty at least three months after SPT placement or failed urethral manipulation. This interval was necessary to allow maturation of the stricture, as earlier intervention may obscure the true extent of fibrosis, leading to underestimation of stricture length and potential intraoperative challenges. All surgeries were performed via perineal approach. An extensive description of the surgical technique is beyond the scope of this paper and our technique of transecting (t-EPA, Fig. 2) and non-transecting EPA (nt-EPA, Fig. 3) has been previously described [24]. At our center, nt-EPA was always attempted as first surgical approach for posterior stenoses when feasible, in line with its original use for bulbar strictures [25]. While t–EPA was reserved for cases where it was not technically feasible to remove all fibrotic tissue adequately or to achieve a tension-free anastomosis with nt-EPA. In patients with a preoperative SPT, intraoperative cystoscopy via the suprapubic tract using a flexible cystoscope was routinely performed when the bladder neck was not visualized radiographically—or more usefully, when it appeared open on RUG/VCUG—to assess bladder neck integrity and function, identify the proximal urethral stump, guide dissection, and, when necessary, facilitate guidewire insertion. In patients without a SPT, spontaneous voiding confirmed urethral patency, and a guidewire was routinely introduced at the beginning of the procedure; when this was not feasible, intraoperative cystoscopy was performed to facilitate guidewire placement. The need for auxiliary maneuvers—such as splitting of the corpora cavernosa, inferior pubectomy, or supracrural rerouting—was determined intraoperatively at the surgeon’s discretion to achieve adequate exposure and a tension-free anastomosis. If SPT was present, it was removed at the end of the surgery while the urethral catheter remained in place for 2 weeks until evaluation for urinary leakage by urethrography. At this stage, the decision to remove or prolong catheterization was based on the presence of complications such as wound infection or contrast extravasation and subsequent evaluation ith RUG was done weekly until the a fortiori mentioned problems resolved.

### Statistical analysis

The normality of variable distributions was assessed using the Kolmogorov–Smirnov test. Categorical variables are reported as frequencies and percentages. Continuous variables with a normal distribution are presented as means and standard deviations (SD), whereas non-normally distributed variables are expressed as medians and interquartile ranges (IQR). Differences between groups were analyzed using the Chi-square test or Fisher’s exact test for discrete variables, or Mann-Whitney for continuous variables, as appropriate. The log-rank test, contingency tables, and logistic regression were used to assess the relationship between dependent and independent variables. The RFS rate was estimated using Kaplan-Meier analysis. The hazard ratio (HR) for stenosis recurrence during follow-up was calculated by Cox proportional hazards regression, following univariate analysis by the log-rank test. Statistical analyses were performed using the Statistical Package for the Social Sciences (SPSS; v. 29.0.2.0; IBM, Chicago, USA), with a two-sided significance level set at *p-*value < 0.05.

## Results

A total of 70 patients were eligible for the current study. Patient demographics are summarized in Table 1. A preoperative SPT was in place in most patients (77.1%), with 60% of patients having a preoperative positive urine culture. Eighteen patients (25.7%) had undergone previous urethral manipulations before posterior urethroplasty.

**Table 1:** Patients’ baseline characteristics

Variables	Total (*n* = 70)	Type of EPA		*p* value
		t-EPA (*n* = 53)	nt-EPA (*n* = 17)	
Nr of patients, n (%)	70 (100)	53 (75.7)	17 (24.3)	0.403
Age (years), (IQR)	41.5 (30.0–56.3)	43.0 (31.8–56.8)	41.0 (24.5–60.0)	0.078
Follow-up (months), (IQR)	130 (78.5–178.5)	118.0 (14.5–256.6)	111.0 (5.5–167.8)	0.403
Smoke, n (%)	7 (10.0)	4 (7.5)	3 (17.6)	0.349
OSAS or asthma, n (%)	6 (8.6)	5 (9.4)	1 (5.9)	1.000
Diabetes, n (%)	5 (7.1)	2 (3.8)	3 (17.6)	0.088
Cardiovascular disorders, n (%)	7 (10.0)	5 (9.4)	2 (11.8)	1.000
Hypertension, n (%)	5 (7.1)	3 (5.7)	2 (11.8)	0.597
Presence of preop suprapubic catheter, n (%)	54 (77.1)	42 (79.2)	12 (70.6)	0.513
Positive preop urine culture, n (%)	42 (60.0)	30 (56.6)	12 (70.6)	0.380
Previous interventions, n (%)	18 (25.7)	13 (24.5)	5 (29.4)	0.753
Dilatation/DVIU	6 (8.6)	4	2	0.628
> 1 dilatation/DVIU	6 (8.6)	5	1	1.000
Endoscopic realignment	2 (2.8)	2	0	1.000
Open realignment	1 (1.4)	1	0	1.000
Endoscopic realignment and Sachse urethrotomy	3 (4.3)	1	2	0.144

*IQR* interquartile range; *OSAS* Obstructive sleep apnea syndrome; *tEPA* transecting excision and primary anastomosis; *ntEPA* non-transecting excision and primary anastomosis

**Table 2:** Surgical and postoperative characteristics

Variables	Total (*n* = 70)	Type of EPA		*p* value
		t-EPA (*n* = 53)	nt-EPA (*n* = 17)	
Hospital stay (days), (IQR)	3.0 (2.0–4.0)	3.0 (2.0–4.0)	2.0 (1.0–3.0)	**0.017**
Operation time (min), (IQR)	120.0 (99.8–140.3)	127.5 (99.8–176.0)	110.0 (99.0–125.0)	**0.035**
Stricture length (cm), (IQR)	2.0 (2.0–4.0)	2.3 (2.0–4.0)	2.0 (1.3–3.5)	0.108
Adjunctive procedures, n (%)	33 (47.1)	28 (52.8)	5 (29.4)	0.090
Corporal splitting	33 (47.1)			
Inferior pubectomy	4 (5.7)			
Crural rerouting	1 (1.4)			
Total bladder catheter stay (days), (IQR)	14.5 (13.0–16.0)	15.5 (14.0–20.3.0)	15.0 (13.5–16.0)	0.157
First cystography postop (days), (IQR)	14.0 (12.3–16.0)	14.0 (13.0–16.0)	15.0 (12.0–16.0)	0.105
Nr patients with any extravasation at first postop pRUG, n (%)	8 (11.4)	6 (11.3)	2 (11.8)	1.000
Last cystography postop in patients having an extravasation at first pRUG (days), (IQR)	22.5 (19.3–35.3)	26.0 (19.8–39.8)	20.0 (17.0–23.0)	0.317
Postoperative complications < 90 days, n (%)	15 (21.4)	11 (20.8)	4 (23.5)	1.000
Clavien-Dindo classification, n (%)				
Grade I	8 (11.4)	5 (9.4)	3 (17.6)	
Grade II	7 (10.0)	6 (11.3)	1 (5.9)	
Grade IIIa	0 (0)	0 (0)	0 (0)	
Grade IIIb	0 (0)	0 (0)	0 (0)	
Grade IVa	0 (0)	0 (0)	0 (0)	
Grade IVb	0 (0)	0 (0)	0 (0)	
Grade V	0 (0)			
Recurrence at follow-up, n (%)	11 (15.8)	8 (15.1)	3 (17.6)	1.000
< 1 year, n (%)	9 (12.9)			
1–5 years, n (%)	1 (1.4)			
> 5 years, n (%)	1(1.4)			
Time to recurrence (months), (IQR)	3.5 (2.3–5.8)	3.5 (3.0–5.5)	2.0 (1.5–3.5)	0.621
Type of recurrence				
Anatomical, n (%)	2 (2.9)			
Functional, n (%)	1 (1.4)			
Both, n (%)	9 (12.9)			
The therapy required for recurrence, n (%)				
Sachse urethrotomy	6 (8.6)	4 (7.5)	2 (11.8)	
Re-do urethroplasty	3 (4.3)	3 (5.7)	0 (0)	
Perineostomy	2 (2.9)	1 (1.9)	1 (5.9)	

*IQR* interquartile range; *tEPA* transecting excision and primary anastomosis; *ntEPA* non-transecting excision and primary anastomosis; *pRUG* pericatheter retrograde urethrography

**Table 3:** Subgroup analysis of the recurrence rate during follow-up and the hazard ratio for recurrence in patients who underwent primary urethral repair

Group	*n* (%)	RFS rate at follow-up (%)	Hazard ratio for RFS during follow-up (95% CI)	*p* for the hazard ratio
Overall	70 (100)	77.8		
Subgroup				
Complications < 90 days	14 (20.0)	57.1	4.85 (1.54–15.26)	**0.007**
Presence of preoperative suprapubic catheter	54 (77.1)	69.3	32.99 (0.13–8317.01)	0.215
Extravasation at first pRUG	8 (11.4)	57.1	3.21 (0.87–11.89)	0.081
Persistent extravasation at the second RUG	5 (7.1)	25.0	6.36 (1.69–23.87)	**0.006 **
Previous interventions	18 (25.7)	83.0	0.95 (0.29–3.52)	0.942
Type of urethroplasty (transecting/non-transecting	53 (75.7)/17 (24.3)	78.5/81.9	1.29 (0.34–4.85)	0.710

*CI* confidence interval; *pRUG* pericatheter retrograde urethrography; Statistically significant *p* values are shown in bold.

The surgical and postoperative characteristics are shown in Table 2. The median hospital stay was 3 days (IQR 2.0–4.0), with a median operation time of 120 min (IQR 99.8–140.3). The median stenosis length was 2.0 cm (IQR 2.0–4.0). Among all patients, 75.5% underwent t-EPA, while the remaining patients underwent nt-EPA. In 33 patients (47.1%), ancillary procedures were performed to achieve tension-free anastomosis: in 29 patients, additional corporal splitting was sufficient, 3 patients required additional inferior pubectomy, and 1 patient needed a further suprapubic rerouting. The first pRUG was performed at a median of 14 days (IQR 12.3–16.0), with 11.4% of patients showing mild to severe urine extravasation. Re-evaluation with pRUG was conducted weekly until resolution was observed. In these patients, the final postoperative cystography confirming fistula closure was performed at a median of 22.5 days (IQR 19.3–35.3).

A total of 20% of patients experienced postoperative complications within 90 days of surgery, all of which were classified as low-grade (Clavien-Dindo < III) and managed conservatively with local ice pack application, prolonged bladder catheterization, or antibiotic administration. The overall number of complications in these 15 patients was 18 (some patients had more than one complication), as represented in Fig. 4. After a median follow-up of 130 months (IQR 78.5–178.5), 11 out of 70 patients (15.8%) experienced recurrence. The 1-year, 2-year, and 10-year RFS rates are 85.4%, 83.8%, and 83.8%, respectively. Most recurrences (10 out of 11 cases) occurred within the first year after primary urethroplasty, with a median time to recurrence of 3.0 months (IQR 2.0–5.0). Patients with recurrence were managed with Sachse urethrotomy (*n* = 6, 8.6%), re-do urethroplasty (*n* = 3, 4.3%), or perineostomy (*n* = 2, 2.9%).

Multiple factors may influence the outcome of posterior urethroplasty, so we performed a multivariate analysis to control for confounding variables and better assess the independent effect of each factor, including age, comorbidities, smoking, stricture length, preoperative positive urine culture, presence of a SPT, type of EPA, postoperative complications, and extravasation. In univariate analysis, preoperative SPT was significantly associated with stricture recurrence (*p* = 0.039); however, this association was not confirmed in multivariate analysis (*p* = 0.215). This may be because patients with an SPT often have longer, more severe strictures, higher rates of preoperative positive urine cultures and infection risk and require more complex repairs—all of which may contribute to a higher risk of recurrence. Similarly, previous urethral manipulations, the type of EPA, comorbidities (DM, hypertension), smoking, and stenosis length > 2 cm were not significantly associated with recurrence risk. However, complications within 90 days post-surgery (HR = 4.85, *p* = 0.007) and persistent extravasation at the second RUG (HR = 6.36, *p* = 0.006) were strongly linked to higher recurrence rates. These findings suggest that delayed wound healing and early postoperative complications play a crucial role in stenosis recurrence (Table 3).

In multivariate analysis, smoking (*p* = 0.621), comorbidities (*p* > 0.05), the presence of SPT (*p* = 0.164), previous interventions (*p* = 0.494), stenosis length (*p* = 0.353), urethroplasty type (*p* = 0.732), and positive preoperative urine culture (*p* = 0.515) were not significantly associated with postoperative complications.

Similarly, logistic regression showed that previous interventions (*p* = 0.156) and stenosis length (*p* = 0.384) were not predictors of adjunctive surgical procedures.

Functional outcomes were assessed in 49 patients for erectile function and 61 for urinary continence. ED was common due to pelvic trauma (97.9%), with 2% worsening in the t-EPA group and 16.3% improving post-urethroplasty, with no group differences. De novo UI occurred in 6.6%, and 36.1% reported mild post-urinary dribbling (0–1 safety pad/day) without impacting quality of life.

## Discussion

PFUI-related stenoses present significant challenges in both the acute and delayed settings. The altered pelvic anatomy, extensive fibrosis at the injury site, and the deep location of the posterior urethra make surgical reconstruction particularly complex [14]. In some cases, additional intraoperative manoeuvres are required to achieve a tension-free anastomosis. Unlike strictures in other urethral segments, posterior urethral reconstruction demands extensive experience and a thorough understanding of local anatomy and functional structures, including the prostate and urinary sphincter. Given these complexities, patients should be referred to high-volume, tertiary centers with specialized expertise in urethral surgery. In this study, we present our experience and analyze potential factors influencing surgical outcomes.

### Recurrent stenosis free survival rates and risk factors for recurrence

The success rate of anastomotic urethral repair in major series varies widely, ranging from 63.5 to 95.5% [13, 18, 19, 26, 27]. In our cohort, most recurrences occurred within the first year, with a median time to recurrence of 3 months, aligning with previous findings [19]. The RFS rates were 85.4% at one year, 83.8% at two years, and remained stable at 83.8% at ten years, suggesting durable long-term outcomes. Recurrences are most likely to occur during the early postoperative months, which is a critical period for wound healing. Factors such as infection, hematoma, anastomotic tension, or ischemia resulting from excessive urethral mobilisation can compromise healing and increase the risk of recurrence. In contrast, long-term analyses by Culty et al. [18] reported declining success rates of 63%, 55%, and 43% at 1, 5, and 10 years, respectively, while Corriere et al. [27] found a 63% ss rate at follow-up. These discrepancies may arise from differences in the definition of recurrence across studies and variations in patient populations.

Several factors, including prior interventions and postoperative complications influence the RFS of urethroplasty. While some studies suggest that prior urethral manipulation may increase surgical difficulty due to fibrosis [11, 12, 18, 28–30], we did not identify it as a negative prognostic factor, nor was it associated with recurrence risk in our analysis (*p* = 0.942). This should not justify repeated endoscopic interventions after failed realignment, as they only delay definitive treatment. However, postoperative complications and persistent extravasation significantly increased the risk of restenosis, by approximately 5 and 6 times, respectively. Hematoma, infection and persistent urine extravasation can lead to prolonged inflammation, fibrosis, and impaired tissue healing at the repair site, promoting excessive scar formation and urethral narrowing, which in turn increases the likelihood of restenosis. Therefore, strict follow-up is essential for these patients, as they are at higher risk for early recurrent stenosis. Although the follow-up protocol remains the same as for patients without complications, it is crucial to inform them of the higher risk and advise early medical consultation if symptoms recur.

In our study, the presence of an SPT and common comorbidities (DM, hypertension) did not influence recurrence risk. Despite their potential impact on microvascular circulation and wound healing, our findings align with those in the literature [19].

The timing of urethroplasty is an important consideration. Several studies have investigated whether the timing of posterior urethroplasty influences surgical outcomes, often reporting high complication rates with immediate repair of PFUIs, including a significant risk of restenosis (53–69%), erectile dysfunction (36–44%), and incontinence (5–21%) [31, 32]. Scarberry et al. [13] explored this question in PFUI patients managed acutely with suprapubic tube (SPT) placement, comparing early (≤ 6 weeks) and delayed (≥ 12 weeks) urethroplasty. Early repair was performed when associated injuries were stable, the perineum was soft on rectal examination, and the pelvic fracture permitted lithotomy positioning. Restenosis-free survival (RFS) was slightly lower in the early group (90.9%) compared to the delayed group (100%). However, the two groups were not entirely comparable. The delayed group included patients deemed unfit for early surgery due to unstable pelvic fractures or significant perineal induration—features suggesting more severe trauma and potentially greater urethral involvement. The role of initial endoscopic realignment and the optimal timing of urethroplasty remain subjects of ongoing debate. Early endoscopic realignment can serve as definitive treatment in approximately 37% of patients and may obviate the need for urethroplasty in over half of those who experience recurrence [10]. By promoting early realignment of the urethral ends, this approach may reduce the incidence of stricture formation, delay its onset, and simplify subsequent urethroplasty [28, 33]. However, it is not always feasible and, in case of recurrence, may prolong the clinical course and delay definitive management. In our study, we generally followed standard practice, deferring urethroplasty for at least three months after SPT placement or failed endoscopic realignment. This approach allows for hematoma resolution, prostate descent, scar tissue maturation, and safe patient positioning.

Stricture location plays a crucial role in PFUI repair outcomes. Harraz et al. [34] analyzed 158 patients and found that the position of the proximal bulbar urethra and the presence of pubic arch fractures were independent predictors of failure, regardless of posterior urethral length or location. Similarly, Fu et al. [20] found that strictures located < 3 cm from the bladder neck had a significantly lower success rate than those > 3 cm away (64.6% vs. 90.5%; *p* < 0.001). Deeper stricture locations, especially those closer to the bladder neck, require more extensive dissection and tissue mobilization. These anatomical challenges result in limited surgical exposure and restricted space for urethral manipulation, which may contribute to higher recurrence rates. Pelvic fractures most commonly associated with urethral injuries include straddle fractures involving all four pubic rami, fractures of the inferior pubic ramus with pubic symphysis diastasis, and deep ring fracture-dislocations such as Malgaigne’s fractures [1]. Consequently, pubic arch fractures are frequently observed in PFUI patients. The absence of such fractures on imaging, particularly on cystourethrography, may indicate a less severe injury, which in turn could be associated with reduced fibrosis and better surgical outcomes.

Another key factor is the length of stenosis. Several studies indicate that longer stenoses are associated with a higher risk of restenosis [19, 35, 36]. For instance, Satyagraha et al. [19] identified a stricture length greater than 2 cm as a recurrence risk factor, while Fu et al. [20] reported a threshold of 1.6 cm. Longer stenoses often require more extensive excision of fibrotic tissue, which increases surgical complexity, tension at the anastomosis, and compromises vascularization – factors that contribute to the risk of recurrence. However, in our study, we did not find stenosis length (≥ 2 cm) to be a significant risk factor for recurrence. This may be due to the importance of achieving a tension-free anastomosis, with the ancillary manoeuvres if needed, regardless of stenosis length.

A further point of interest is the impact of a vascular-sparing approach during EPA repair. Preserving the bulbar arteries of the corpus spongiosum has been proposed as a method to reduce the risk of postoperative stenosis recurrence. Gomez et al. [7] suggested that vascular preservation may help prevent restenosis after PFUI reconstruction. However, further comparative and prospective studies are required to validate this hypothesis. In our study, we found no statistically significant difference in recurrence rates between standard and vascular-sparing techniques (*p* = 0.710), suggesting that nt-EPA remains a viable option when feasible. It minimizes damage to the urethral vascularization, which may already be compromised by trauma. Sparing the anatomical blood supply should be encouraged, and non-transecting techniques that preserve the bulbar blood supply are worth attempting, as they do not negatively affect outcomes. In fact, the preserved blood supply may improve the results of subsequent urethral surgeries, such as redo-urethroplasty or incontinence surgery.

### Complication rates and risk factors for complications

Complications associated with EPA repair include hematoma, UTI, wound infection, extravasation at the anastomotic site (with or without urethrocutaneous fistula), and rectal injury. Mathur et al. [37] reported an overall complication rate of 20.3%, while Gomez et al. [7] observed 23%, with only one high-grade event (Clavien III), and 17% of cases requiring prolonged catheterization due to extravasation. In our study, we found a 21.4% complication rate, all classified as low-grade (Clavien < III). Although classified as minor, such complications may negatively impact urethroplasty outcomes. Postoperative local extravasation requiring prolonged catheterization and hematoma formation can impair wound healing, create an unfavorable local environment, and lead to persistent infection or even abscess formation, resulting in prolonged inflammation and excessive scar formation. We did not identify any significant correlation between smoking, comorbidities, preoperative positive urine culture, SPT presence, previous interventions, stenosis length, or EPA repair type and complication risk. These findings suggest that while complications are common, they are mostly low-grade and manageable, with no major complications requiring significant intervention.

### Functional outcomes

The impact of perineal urethroplasty on erectile function remains debated. In our cohort, 97.9% of patients experienced ED after PFUI, primarily due to nerve damage, though other causes cannot be excluded (vasculogenic and mixed) [5]. Our findings suggest that midline dissection and stepwise tension-free anastomosis have minimal effects on potency. Only 2.2% developed de novo ED, while 16.3% reported improvement after urethroplasty. These outcomes align with other studies showing recovery of erectile function in up to 20% of patients after urethroplasty [1, 26]. Overall, posterior urethroplasty does not appear to be a primary cause of ED. Instead, erectile function seems more closely linked to the severity of the pelvic fracture. Literature reports ED following PFUI in 44%-72% of cases, with de novo ED after urethroplasty around 4% [5, 13, 15, 28].

UI after urethral reconstruction for PFUI is relatively uncommon. Our incidence of de novo UI (6.6%) is consistent with the reported range of 2.1%-7.7% in the literature [13, 28]. Assessing urinary function in PFUI patients is challenging due to the frequent occurrence of acute urinary retention, particularly in cases of complete or partial urethral rupture, necessitating SPT placement. VCUG helps evaluate bladder neck function, and an open bladder neck may predict postoperative UI [38]. However, UI can also occur in cases with a closed bladder neck, as PFUI may cause direct injury to the external urethral sphincter at the bulbo-membranous urethra or damage the pelvic nerves, leading to neurological dysfunction, or result in external sphincter injury during urethral reconstruction, particularly due to scar tissue excision at the membranous urethra. Continence is typically preserved in these patients through an effective internal (proximal) sphincter mechanism, supported by the supramontanal prostatic urethra and bladder neck [39]. As such, future interventions like prostatectomy or bladder neck incision may lead to stress UI, which should be discussed with patients.

### Study limitations

This study has several limitations. Despite being prospectively collected, data were analyzed retrospectively, potentially introducing biases in patient selection and data completeness. Initial management was not always performed at our referral center, affecting care consistency. The lack of trauma type data limits outcome assessment, and subjective functional outcome measures, such as erectile dysfunction and urinary incontinence, may introduce bias. The use of standardized questionnaires would have improved reliability. Lastly, the small cohort limits statistical power and generalizability.

## Conclusion

This study highlights the high long-term RFS with relatively low reintervention rate (15.8%) associated with posterior urethroplasty in patients with PFUI. Stenosis recurrence was associated with postoperative complications and persistent extravasation, highlighting the importance of close follow-up during the first year. Importantly, the use of a non-transecting approach, when anatomically feasible, does not appear to compromise surgical outcomes. Functional outcomes were consistent with existing literature: ED was primarily trauma-related and did not vary by surgical technique, with a low rate of de novo cases (2.2%) and some improvement over time (16.3%), suggesting potential for recovery. Urinary continence was largely preserved, with low rates of de novo incontinence (6.6%) and mild post-void dribbling not affecting quality of life.

## Supplementary Information

Below is the link to the electronic supplementary material.

Supplementary Material 1

Supplementary Material 2

## Data Availability

No datasets were generated or analysed during the current study.

## References

[1] Barratt RC, Bernard J, Mundy AR, Greenwell TJ (2018) Pelvic fracture urethral injury in males—mechanisms of injury, management options and outcomes. Transl Androl Urol 7:S29–S62 29644168 10.21037/tau.2017.12.35 PMC5881191

[2] Johnsen NV, Kaufman MR, Dmochowski RR, Milam DF (2018) Erectile dysfunction following pelvic fracture urethral injury. Sex Med Rev 6:114–123 10.1016/j.sxmr.2017.06.004 28757357

[3] Ballesteros Ruiz C, Toribio-Vázquez C, Fernández-Pascual E, Ríos E, Rodríguez Serrano A, Alonso Dorrego JM, De Girón M, Moreno JA (2022) Cárcamo Valor P, Martínez-Piñeiro L. Repair of Traumatic Urethral Strictures: La Paz University Hospital Experience. JCM 12:54 10.3390/jcm12010054 PMC9821198 36614853

[4] Clark SS, Prudencio RF (1972) Lower urinary tract injuries associated with pelvic fractures: diagnosis and management. Surg Clin North Am 52:183–201 5013219 10.1016/s0039-6109(16)39642-6

[5] Shenfeld OZ, Kiselgorf D, Gofrit ON, Verstandig AG, Landau EH, Pode D (2003) The incidence and causes of erectile dysfunction after pelvic fractures associated with posterior urethral disruption. J Urol 169:2173–2176 12771742 10.1097/01.ju.0000067660.51231.05

[6] Webster GD, Goldwasser B (1986) Perineal transpubic repair: a technique for treating Post-Traumatic prostatomembranous urethral strictures. J Urol 135:278–279 3944858 10.1016/s0022-5347(17)45609-6

[7] Gómez RG, Velarde LG, Campos RA, Massouh R, Humerez V, Barrientos V (2025) Outcomes of bulbar artery sparing during anastomotic urethroplasty for pelvic fracture urethral injury. Actas Urológicas Españolas (English Edition) 49:102–107 10.1016/j.acuroe.2024.11.005 39617177

[8] Xie H, Li C, Xu Y-M, Feng C, Lv X-G, Chen L, Li H-B, Xue J-D (2017) Preliminary experience of nontransecting urethroplasty for pelvic Fracture-related urethral injury. Urology 109:178–183 28735015 10.1016/j.urology.2017.07.013

[9] Horiguchi A, Shinchi M, Masunaga A, Okubo K, Kawamura K, Ojima K, Ito K, Asano T, Azuma R (2017) Primary realignment for pelvic fracture urethral injury is associated with prolonged time to urethroplasty and increased stenosis complexity. Urology 108:184–189 28606774 10.1016/j.urology.2017.06.001

[10] Johnsen NV, Dmochowski RR, Mock S, Reynolds WS, Milam DF, Kaufman MR (2015) Primary endoscopic realignment of urethral disruption Injuries—a double-edged sword?? J Urol 194:1022–1026 25849600 10.1016/j.juro.2015.03.112

[11] Firmanto R, Irdam GA, Wahyudi I (2016) Early realignment versus delayed urethroplasty in management of pelvic fracture urethral injury: a Meta-analysis. Acta Med Indones 48 27550878

[12] Chaker K, Bibi M, Ouanes Y, Chedly WB, Rahoui M, Dali KM, Nouira Y (2023) Comparison of long-term results according to the primary mode of management of injury for posterior urethral injuries. Int Urol Nephrol 55:1971–1975 37249727 10.1007/s11255-023-03648-4

[13] Scarberry K, Bonomo J, Gómez RG (2018) Delayed posterior urethroplasty following pelvic fracture urethral injury: do we have to wait 3 months? Urology 116:193–197 29545047 10.1016/j.urology.2018.01.018

[14] Gomez RG, Scarberry K (2018) Anatomy and techniques in posterior urethroplasty. Transl Androl Urol 7:567–579 30211047 10.21037/tau.2018.03.05 PMC6127558

[15] Flynn BJ, Delvecchio FC, Webster GD (2003) Perineal repair of pelvic fracture urethral distraction defects: experience in 120 patients during the last 10 years. J Urol 170:1877–1880 14532797 10.1097/01.ju.0000091642.41368.f5

[16] Webster GD, Ramon J (1991) Repair of pelvic fracture posterior urethral defects using an elaborated perineal approach: experience with 74 cases. J Urol 145:744–748 2005693 10.1016/s0022-5347(17)38442-2

[17] Yepes C, Oszczudlowski M, Joshi PM, Anand A, Bhadranavar S, Kulkarni SB (2024) Predictors of elaborated perineal or a combined abdominoperineal approach during repair for pelvic fracture urethral injury. World J Urol 42:40 38244107 10.1007/s00345-023-04733-0

[18] Culty T, Boccon-Gibod L (2007) Anastomotic urethroplasty for posttraumatic urethral stricture: previous urethral manipulation has a negative impact on the final outcome. J Urol 177:1374–1377 17382735 10.1016/j.juro.2006.11.092

[19] Satyagraha P, Wibowo E, Daryanto B, Duarsa GWDP, Wijaya AG, Dhani FK (2025) Ten years’ single surgeon experience of excision and primary anastomosis urethroplasty for traumatic urethral stricture: an analysis of risk factors for urethral stricture recurrence. Arch Ital Urol Androl 10.4081/aiua.2025.13268 39851052

[20] Fu Q, Zhang Y, Barbagli G, Zhang J, Xie H, Sa Y, Jin S, Xu Y (2015) Factors that influence the outcome of open urethroplasty for pelvis fracture urethral defect (PFUD): an observational study from a single high-volume tertiary care center. World J Urol 33:2169–2175 25774006 10.1007/s00345-015-1533-4 PMC4655004

[21] Neu S, Remondini T, Hird A, Locke JA, Herschorn S, Kodama R (2022) A retrospective look at term outcomes after definitive surgical repair for traumatic pelvic fracture urethral Injuries—does initial management make a difference?? Urology 160:203–209 34843746 10.1016/j.urology.2021.10.036

[22] Clavien PA, Barkun J, De Oliveira ML, Vauthey JN, Dindo D, Schulick RD, De Santibañes E, Pekolj J, Slankamenac K, Bassi C, Graf R, Vonlanthen R, Padbury R, Cameron JL, Makuuchi M (2009) The clavien-dindo classification of surgical complications: five-year experience. Ann Surg 250 10.1097/SLA.0b013e3181b13ca2 19638912

[23] Chapman DW, Cotter K, Johnsen NV, Patel S, Kinnaird A, Erickson BA, Voelzke B, Buckley J, Rourke K (2019) Nontransecting techniques reduce sexual dysfunction after anastomotic bulbar urethroplasty: results of a Multi-Institutional comparative analysis. J Urol 201:364–370 30266331 10.1016/j.juro.2018.09.051

[24] Lumen N, Hoebeke P, Troyer BD, Ysebaert B, Oosterlinck W (2009) Perineal anastomotic urethroplasty for posttraumatic urethral stricture with or without previous urethral manipulations: a review of 61 cases with Long-Term followup. J Urol 181:1196–1200 19152939 10.1016/j.juro.2008.10.170

[25] Verla W, Oosterlinck W, Waterloos M, Lumen N (2019) Vessel-sparing excision and primary anastomosis. J Vis Exp 10.3791/58214 30663665

[26] Koraitim MM (1995) The lessons of 145 posttraumatic posterior urethral strictures treated in 17 years. J Urol 153:63–66 7966793 10.1097/00005392-199501000-00024

[27] Corriere JN (2001) 1-stage delayed bulboprostatic anastomotic repair of posterior urethral rupture: 60 patients with 1-year followup. J Urol 165:404–407 11176383 10.1097/00005392-200102000-00012

[28] Zou Q, Zhou S, Zhang K, Yang R, Fu Q (2017) The immediate management of pelvic fracture urethral Injury—Endoscopic realignment or cystostomy? J Urol 198:869–874 28442385 10.1016/j.juro.2017.04.081

[29] Elshout PJ, Veskimae E, MacLennan S, Yuan Y, Lumen N, Gonsalves M, Kitrey ND, Sharma DM, Summerton DJ, Kuehhas FE (2017) Outcomes of early endoscopic realignment versus suprapubic cystostomy and delayed urethroplasty for pelvic Fracture-related posterior urethral injuries: a systematic review. Eur Urol Focus 3:545–553 28753868 10.1016/j.euf.2017.03.001

[30] Singh BP, Andankar MG, Swain SK, Das K, Dassi V, Kaswan HK, Agrawal V, Pathak HR (2010) Impact of prior urethral manipulation on outcome of anastomotic urethroplasty for Post-traumatic urethral stricture. Urology 75:179–182 19854488 10.1016/j.urology.2009.06.081

[31] Koraitim MM (1996) Pelvic fracture urethral injuries: evaluation of various methods of management. J Urol 156:1288–1291 8808856 10.1016/s0022-5347(01)65571-x

[32] Cooperberg MR, McAninch JW, Alsikafi NF, Elliott SP (2007) Urethral reconstruction for traumatic posterior urethral disruption: outcomes of a 25-year experience. J Urol 178:2006–2010 17869302 10.1016/j.juro.2007.07.020

[33] Koraitim MM (2012) Effect of early realignment on length and delayed repair of postpelvic fracture urethral injury. Urology 79:912–915 22342415 10.1016/j.urology.2011.11.054

[34] Harraz AM, Nabeeh A, Elbaz R, Abdelhamid A, Tharwat M, Elbakry AA, El-Hefnawy AS, El-Assmy A, Mosbah A, Zahran MH (2023) Could the bulbar urethral end location on the cystourethrogram predict the outcome after posterior urethroplasty for pelvic fracture urethral injury? Arab J Urol 21:94–101 37234680 10.1080/2090598X.2022.2138119 PMC10208150

[35] For the Trauma Urologic Reconstruction Network of Surgeons(TURNS), Johnsen NV, Moses RA, Elliott SP, Vanni AJ, Baradaran N, Greear G, Smith TG, Granieri MA, Alsikafi NF, Erickson BA, Myers JB, Breyer BN, Buckley JC, Zhao LC, Voelzke BB (2020) Multicenter analysis of posterior urethroplasty complexity and outcomes following pelvic fracture urethral injury. World J Urol 38:1073–1079 31144093 10.1007/s00345-019-02824-5

[36] Topaktaş R, Ürkmez A, Tokuç E, Akyüz M, Kutluhan MA (2019) Hematologic parameters and Neutrophil / Lymphocyte ratio in the prediction of urethroplasty success. Int Braz J Urol 45:369–375 30785704 10.1590/S1677-5538.IBJU.2018.0682 PMC6541144

[37] Mathur R, Aggarwal G, Satsangi B, Khan F, Odiya S (2011) Prognosis of urethral strictures following pelvic fracture urethral distraction defects–a single centre study. Int J Surg 9:68–71 20887822 10.1016/j.ijsu.2010.09.004

[38] Iselin CE, Webster GD. The significance of the open bladder neck associated with pelvic fracture urethral distraction defects. no date 10411036

[39] Bagga HS, Angermeier KW (2015) The mechanism of continence after posterior urethroplasty. Arab J Urol 13:60–63 26019981 10.1016/j.aju.2014.11.006 PMC4434879

