# Supplementary material for: Posterior urethroplasty for pelvic fracture urethral injuries: risk factors for recurrence and complications
Source: World J Urol. 2025 Aug 1;43(1):469. doi: 10.1007/s00345-025-05839-3 (PMC12316716; doi:10.1007/s00345-025-05839-3)
Supplement: Supplementary file 2 — Supplementary Material 2 [file 345_2025_5839_MOESM2_ESM.docx]

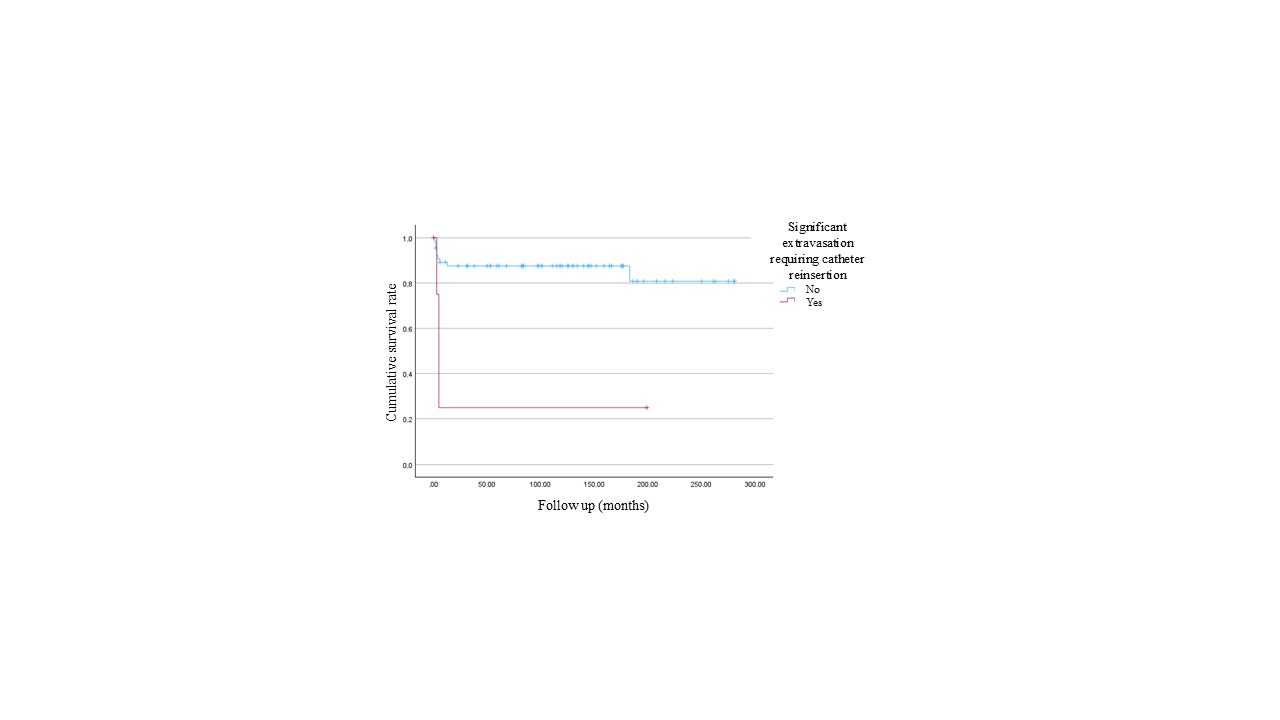


Figure 3. Kaplan-Meier plot of recurrence during follow-up in patients with and without persistent extravasation at second RUG
